# “Boring” family routines reduce non-communicable diseases: a commentary and call for action

**DOI:** 10.1186/s13690-015-0077-9

**Published:** 2015-07-01

**Authors:** Mary Jane Rotheram-Borus, Mark Tomlinson, Emily Davis

**Affiliations:** Department of Psychiatry and Biobehavioral Sciences, Semel Institute, University of California at Los Angeles, 10920 Wilshire Blvd., Suite 350, Los Angeles, CA 90024 USA; Department of Psychology, Stellenbosch University, Private Bag X1, Matieland Stellenbosch, 7602 South Africa

**Keywords:** Non-communicable diseases, NCD, Routines, Family wellness

## Abstract

As global donors shift their efforts from infectious diseases to non-communicable diseases (NCD), it is critical to capitalize on our prior mistakes and successes. Policy makers and public health administrators are often looking for magic bullets: drugs or treatments to eradicate disease. Yet, each potential magic bullet requires consistent, daily implementation and adherence to a new set of habits to actually work. Families’ and communities’ daily, interlocking routines will be the battlefield on which scientific and technological breakthroughs will be implemented and succeed or not.

Currently, there are many evidence-based interventions (EBI) which have been demonstrated to shift specific habits which account for most NCD (eating, drinking, moving, and smoking). Yet, securing sustained uptake of these programs is rare – suggesting different intervention strategies are needed. Structural changes, policy nudges, and partnerships with private enterprise may be able to shift the health behaviors of more citizens faster and at a lower cost than existing EBI. Addressing concurrent risk and protective factors at the community level and intervening to shape new cultural routines may be useful to reduce NCD.

## Background

Non-communicable diseases (NCD) account for a growing public health burden exceeding all communicable, maternal and perinatal nutrition-related deaths combined [1,2]. In most high income countries, NCD account for more than 80% of deaths [3], and still the World Health Organization (WHO) predicts that NCD deaths will increase by 17% worldwide over the next decade [1]. NCDs are also saturating health care needs in low and middle income countries (LMIC), with 80% of all NCD deaths occurring in LMIC [1,3,4].

In September 2011, the United Nations convened a high level meeting on the Prevention and Control of Non-Communicable Diseases with a focus on four conditions that together account for more than 50% of all deaths in LMIC – cancer, heart disease, diabetes and respiratory disease [1,3]. Funding streams in the last three decades have, for the most part, narrowly targeted infectious diseases such as HIV, malaria, and TB [5] – but this investment is shifting. In this commentary and call for action, we argue that as global donors shift funding to support NCD [1] they avoid adopting the intervention strategies utilized to fight infectious diseases. There has been an overemphasis on the scientific search for magic bullets – medications and technological innovations [6]. This certainly has been the case for HIV; with antiretroviral therapies offering lengthened, high quality life, but, with lifelong adherence a necessity, the habits that allowed HIV to become a generalized epidemic remain and continue to result in high HIV incidence (e.g., South Africa [7]), despite the broad availability of drugs. This commentary encourages donors and public health officials to examine the basic processes that sustain both NCD and infectious diseases before investing in a new set of magic bullet solutions.

## Routines of daily living

For the last 25 years, about two-thirds of deaths globally (36 million deaths annually) have been the result of non-communicable diseases (NCD) [1,4]. A third of NCD deaths in LMIC are among people younger than 60 years [3,4], while NCD deaths are over 60 years old in high income countries [1]. These premature deaths and diseases are largely preventable by the implementation of effective interventions that address reductions in risk factors (tobacco, excessive alcohol use, poor diet, physical inactivity, high blood pressure) and enable health systems to effectively respond [1].

There is preliminary evidence demonstrating that the current NCD intervention strategies are not working: for example, failures are mounting for tobacco control in LMIC, treatment for persons at high risk for cardiovascular diseases and multi-sectorial responses to NCD [8]. Current health care responses to NCD are focused on looking for “disease,” rather than on preventing NCD by supporting prosocial patterns of daily living [9]. Four habits account for about half of all global morbidity and mortality: how much and what we eat, exercise, smoking, and alcohol use [4,10]. If the habits of sleeping, mating, and our daily relationships are taken into account, substantially more of our health and well-being can be improved [11,12]. One domain, critically implicated in the growth of NCD and that receives very little research attention, is that of the routine habits of daily family life.

Sustained reductions in NCD depend on small shifts in families’ and communities’ habits. Reducing caloric intake each day by a small amount may result in weight loss of 9 kg annually (e.g., eating one less apple daily) [13]. Regular family meals improve health and overall well-being [14], and create opportunities for family bonding and discussions about the day’s activities. Preschool-aged children in the United States exposed to the three household routines - regularly eating dinner as a family, getting adequate nighttime sleep, and having limited screen-viewing time - have a 40% lower prevalence of obesity than those exposed to none of these routines [15]. There are rhythmic patterns to the day in almost all countries, with few variations. Among 10 European countries, people spend about 2.5 hours on meals, four hours on household chores, four hours working and have 4–5 hours of free time per day [16]. In contrast, the typical American spends only about an hour primarily focused on eating meals and another 1.5 hours drinking (other than water), typically while engaged in an activity such as watching television, driving, or working [17]. Another 10% of American children’s meals are eaten at McDonalds [18]. Americans have less time for meals and chores, and spend more time working [19]. These are the habits of daily living that must be addressed in order to improve NCD.

When focusing on habits, the co-dependency among family members is immediately evident [20]. Changing a single behavior of one family member triggers a cascade of changes in associated habits, which are similarly embedded in synchronized multi-person routines– to either increase or reduce NCD [21]. Habits are embedded in family members’ lives at home, at school, and in community activities. It is in these contexts that one must establish and maintain healthy habits to prevent NCD. While we are not suggesting that prevention and treatment are mutually exclusive, for people with a NCD, a nuanced combination of prevention and treatment is needed.

## Discussion

### Context matters

In creating the next generation of interventions, context matters. Our intervention programs need to recognize the contexts of the families in their communities and must shape and nudge families into acting and feeling healthier [22]. Globally, it is private entrepreneurs, not health and public health advocates, who have shaped families' daily routines [23]. In a high income country, such as the United States, television, internet, mobile phones, video games, and social media now occupy each family member for about 4–5 hours daily, and about 40,000 commercials are viewed annually [23]. Active, engaged lifestyles are likely to require partnerships with private enterprise to reshape these family patterns.

Families perceive their health to be under their own control [24]. Yet, families’ habits largely reflect where they live, who their friends are, and what they watch on TV. The environments in which we live determine our opportunities and the pulls for healthy and unhealthy behaviors - a built environment with sidewalks can build active lifestyles and friendships [25]. The density of fast food restaurants influences food choices and is often higher in low income neighborhoods compared to high income neighborhoods [26]. Most families in a community face the same set of health challenges, yet families are likely to attribute responsibility to unhealthy behaviors or a lack of willpower, not perceiving the influence of environmental conditions [24]. Thus, the features of the local community either heighten risk or protect families from NCD. Nudging families to change their daily routines will require structural support (i.e., large-scale interventions) in the form of legislative and administrative policies.

The potential benefits of healthy family routines for reducing NCD are significant and substantial. Similar to having family meals, each of the habits listed below has been consistently associated with better health and has benefits that extend far beyond the activities themselves:

- Having an active lifestyle and engaging in sports activities and exercise [27];
- Sleeping at least seven hours a day [28,29];
- Monitoring children’s whereabouts and activities [30];
- Eating fruits and vegetables, rather than processed, sugary foods [24], which has the added benefits of reducing women’s propensity to smoke cigarettes to control their weight [31]; and
- Maintaining consistent routines over sustained periods of time [20].

Each of these actions embeds the family in a network of like-minded peers. Playing sports surrounds children with peers whose families also have active lifestyles [27]. Having dinner or bedtime at a regular time creates a culture of predictability in a family. Thus, each action has a broader and deeper developmental impact than the act itself (sleep, eating, exercising). These behaviors are not the types which respond to magic bullets, nor are they likely to be shaped by interactions with a health care provider (who may see a family a two-three times annually). Interlocking habits of family members can be only be shaped towards increasing health slowly over time with small steps which are mutually reinforcing among family members and friends [32].

## Way forward and a call for action

Structural interventions are needed to provide families with opportunities to optimize their health. Currently, we typically reshape families’ habits by mounting evidence-based interventions (EBI) which target single outcomes. These EBI are labor intensive, time-limited, and delivered in individual sessions or small group meetings at community sites [33,34]. Adding specialized meetings to a family’s existing daily routine is unlikely to be sustainable over the long-term. The opportunities for change need to be embedded in families’ existing schedules.

Throughout China, parents, the elderly and young people can be found at 6 a.m. in the streets: dancing, doing Tai Chi, meeting in exercise groups, or socializing while walking. This routine is part of each person’s day, creating a culture that supports vigorous activity daily. In the United States, some elementary and high schools have started offering Zumba on the playground, as children arrive, waiting for school to begin so that a potentially inactive period is substituted with vigorous dancing [32]. Activity, such as Zumba, helps teachers manage classrooms more easily and primes children’s attention [32]. Cultural routines such as these create opportunities for reducing NCD; TV watching eliminates healthy opportunities.

Structural interventions create the opportunities to improve family routines, not by slowly shaping new behaviors, but by changing the incentives or choices that families have [35-37]. For example, taxing tobacco and alcohol reduces use significantly and reductions are sustained over time [38,39]. Controlling alcohol advertising also reduces alcohol use, especially among adolescents [40]. Taxing cigarettes and sugary soda drinks, and raising the legal drinking age are additional examples of structural interventions which significantly reduce risk patterns related to NCD [38,41,42]. Government agencies often require improvements in food content, changing the quality of food families eat [43]. For example, with the availability of farmers’ markets, families can more easily shift their diet from processed food to raw fruits and vegetables. The number of farmers’ markets in the United States has grown fourfold since 1994 and 180% from 2006–2014 [44,45]. The European Union formally encourages fresh produce and local crops with a “Farm to Fork” initiative [46]. Each of these distal structural policies shapes families’ proximal routines and realigns incentives to encourage families to maintain the habits. The Robert Wood Johnson Foundation has initiated this type of approach in its commitment to a *Culture of Health*, which emphasizes policy-level shifts that incentivize healthy acts [47].

These structural shifts affect more people in a shorter period of time at lower cost than would typically be achieved with our existing approaches to delivering EBI. The business world refers to such interventions as “disruptive” innovations [48,49]. When we embed opportunities for health into families’ days, we may be improving the health of more people faster [50], a possibility which will require substantial revision to the way scientists approach the creation and diffusion of EBI [24].

Funding streams drive programs and the uptake of health interventions. Donor agencies invested in infectious diseases over the last 20 years targeted a single health outcome – typically HIV, TB, or malaria [51-53]. While creating access to services, having specialized sites for HIV-related service functioned to increase the stigma of the disease and HIV care [54]. Programs to reduce NCD are likely to be more successful if multiple behaviors, both risk and protective factors, are targeted within a single site, i.e., if programs are horizontally integrated. Continuing to organise funding streams, staffing, intervention programs, and populations based on their targeted NCD (a vertically siloed program) will waste substantial resources and expertise that could be integrated to form a network of support for the full range of families’ risk and protective factors.

## Conclusion

Now is the time to ensure that we do not continue the mistakes of the last two decades. Blockbuster drugs, vertically integrated systems to beat cardiovascular disease and diabetes, and preventive surgeries are not solutions to the root causes of NCD. Our research agenda and donor investments need to shift away from existing focus. Family wellness, optimized daily with good habits, is the only sustainable, long-term solution to NCD. We advocate in this call for action that policy-makers and researchers focus on structural mechanisms which can be broadly diffused by:

- Creating structural opportunities for families to create healthy daily routines, in their habitual activities, food and drink choices, sleep, and work/school schedules;
- Integrating funding streams across different NCD, so that the primary health threats within a specific community context are addressed; and,
- Empirically examining how community shifts in the risk and protective factors for multiple diseases either efficaciously changes health over the long-term (or not).
